# Osteoclast-derived coupling factors: origins and state-of-play Louis V Avioli lecture, ASBMR 2023

**DOI:** 10.1093/jbmr/zjae110

**Published:** 2024-07-11

**Authors:** Natalie A Sims

**Affiliations:** Bone Cell Biology and Diease Unit, St. Vincent’s Institute of Medical Research, Fitzroy, Victoria 3065, Australia; Department of Medicine at St. Vincent’s Hospital Melbourne, The University of Melbourne, Fitzroy, Victoria 3065, Australia; The Mary McKillop Institute for Health Research, Australian Catholic University, Fitzroy, Victoria 3065, Australia

**Keywords:** coupling, remodeling, osteoclast, osteoblast, osteocyte

## Abstract

Coupling, the mechanism that controls the sequence of events in bone remodeling, is a fundamental theory for understanding the way the skeleton changes throughout life. This review is an adapted version of the Louis V Avioli lecture, delivered at the Annual Scientific Meeting of the American Society of Bone and Mineral Research in 2023. It outlines the history of the coupling concept, details how coupling is thought to occur within trabecular and cortical bone, and describes its multiple contexts and the many mechanisms suggested to couple bone-forming osteoblasts to the prior action of osteoclasts on the same bone surface. These mechanisms include signals produced at each stage of the remodeling sequence (resorption, reversal, and formation), such as factors released by osteoclasts through their resorptive action and through protein synthesis, molecules deposited in the cement line during the reversal phase, and potential signals from osteocytes within the local bone environment. The review highlights two examples of coupling factors (Cardiotrophin 1 and EphrinB2:EphB4) to illustrate the limited data available, the need to integrate the many functions of these factors within the basic multicellular unit (BMU), and the multiple origins of these factors, including the other cell types present during the remodeling sequence (such as osteocytes, macrophages, endothelial cells, and T-cells).

## Introduction

### Basic concepts: Coupling occurs in bone remodeling

Coupling is a fundamental process that underlies our understanding of bone biology. This concept developed as a theory to explain the sequence of events in bone remodeling, conducted by multiple cell types, termed the basic multicellular unit (BMU).

**Figure 1:**
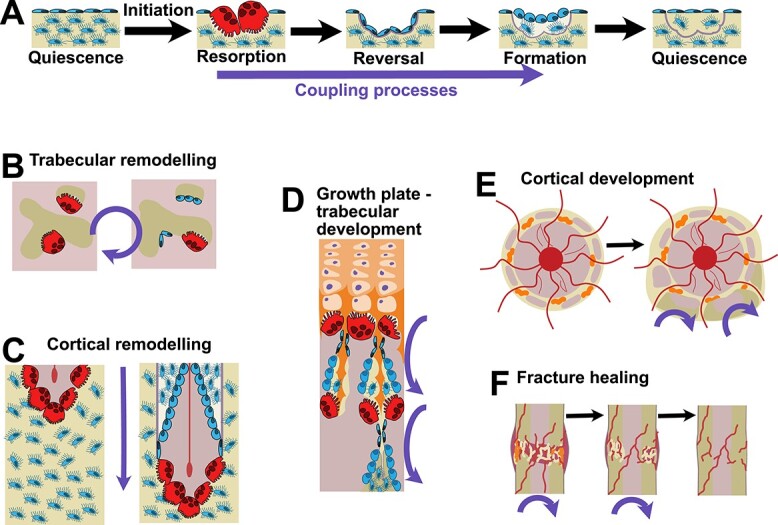
The bone remodeling sequence, highlighting the duration of coupling processes (A), and their occurrences in adult trabecular (B) and cortical (C) remodeling, in trabecular (D) and cortical (E) bone development, and in fracture healing. During the remodeling sequence (A), after initiation at a quiescent bone surface, the sequence of resorption, reversal, and formation occurs, with coupling processes responsible for maintaining this sequence of events during the period highlighted under the sequence. This sequence of events occurs in multiple contexts. In trabecular bone remodeling (B), it occurs on the bone surfaces in contact with the marrow space. In cortical remodeling (C), the sequence occurs in a cutting cone that tunnels into the bone matrix, replacing old bone with new on top of the cement line. During development of trabecular bone (D), the same sequence replaces mineralized growth plate cartilage with woven bone and repeats to replace woven bone with lamellar bone. In cortical development (E), the porous woven template, which contains cartilage is replaced, by the same sequence, with lamellar bone. During fracture healing (F), the initial deposits of woven bone and cartilage are gradually replaced with robust lamellar cortical bone by the same sequence of events. See text for further details.

Bone remodeling is the process where bone resorption is followed by bone formation on the same surface of bone. It is generally understood to be a maintenance process that renews the skeleton, responding to tissue damage, and making calcium available to the circulation, but it has many more functions than this (Figure 1).

The term “remodeling” has evolved to become distinct from “modeling.” Although early papers (eg, Enlow^1^^,^^2^) used these terms in multiple ways, we will use these terms as defined by Frost^3^ to describe cellular activities on bone surfaces, as follows. In remodeling, bone formation follows resorption of bone on the same surface. In modeling, bone formation and bone resorption occur on different surfaces and are not sequential.^4^ This separation of activities in modeling leads to changes in bone size and shape. One example of modeling is periosteal expansion during diametric growth. This process occurs on the two surfaces separated by the cortical bone: bone formation occurs at the periosteum, while bone resorption occurs at the endocortical surface, leading to wider bones while maintaining cortical thickness.^2^

Coupling is restricted to the process of bone remodeling because it is the control mechanism determining the sequence of cellular events.^5^^,^^6^ The remodeling sequence was first described based on histology of adult human trabecular and cortical bone (Figure 1A).^5^^,^^6^ These activities could be inferred based on histology, because bone keeps an historical record of past events. Frost and colleagues, initially describing trabecular bone, noted that the scalloped shape of resorptive surfaces, known as Howship’s lacunae, was preserved in the shape of the cement line underlying the smooth surfaces remaining after bone formation had occurred.^6^ They proposed that the scalloped shape of the cement line reflected the prior activity of osteoclasts, meaning that bone resorption must have occurred prior to bone formation on this surface. A smooth bone surface with an underlying resorbed surface could therefore be defined as a bone surface that had undergone remodeling. They observed that the vast majority (97%) of trabecular bone showed evidence of having undergone this sequence (Figure 1B).

Just a month later, Frost noted the same sequence of events in cortical bone.^5^ In this context, the remodeling sequence commenced with a cutting cone (Figure 1C). Again, based on histology, Frost noted five processes that moved through the bone “as a coherent group” of activities. First, osteoclasts form the blunt-pointed end of the cone by resorbing a tunnel through the bone. This was followed by dividing spindle cells and blood vessels, a period which later became known as the reversal phase. Osteoblasts then appeared on the same surface formed by prior osteoclastic resorption to form new bone. This final action of the osteoblasts gradually refilled the tunnel, leaving the central vascular canal. Key to these observations were that the five processes moved through the bone together in sequence. This implied a “coupling” mechanism by which osteoclasts must signal to the subsequent cells, bringing them to the bone surface where the osteoclasts had previously acted.

### Basic concepts: Remodeling occurs in multiple contexts

Although bone remodeling is most often considered in adult bone, where it renews and restores mechanical integrity (Figure 1A and B), the same process also occurs during endochondral ossification in bone development and bone growth (Figure 1D). For example, during bone growth, bone remodeling occurs sequentially in the primary spongiosa, an anatomically defined area in the metaphysis, adjacent to the growth plate.^7^ Osteoclasts are observed in histological sections resorbing the calcified cartilage surrounding hypertrophic chondrocytes.^8^ This is followed by the same sequence of activities previously observed within the cutting cone. Here, the sequence can be observed histologically with increasing distance from the hypertrophic zone^7^: after osteoclasts depart the resorbed calcified cartilage surface, osteoblasts appear and form woven bone on that surface.^9^ There then follows a second sequence of bone remodeling,^7^ with osteoclasts again being followed by osteoblasts, which in this case form lamellar bone.^9^ This same sequence therefore occurs in the contexts of bone development, longitudinal bone growth, and the development of trabecular structures.

The same sequence of events also occurs during cortical bone development (reviewed in Koh et al.^10^). Here, calcified cartilage remnants in the cortical bone collar are resorbed and replaced with woven bone on the same surface (Figure 1E). As at the growth plate, this woven bone is resorbed by osteoclasts and replaced with consolidated primary lamellar bone.^1^ In larger animals, this primary lamellar bone continues to be remodeled by initiation of cutting cones, resulting in formation of primary osteons.^11^ With continuing remodeling, secondary osteons interrupt the borders of these cutting cones. Remodeling of the cortical structure continues throughout life, as described above.

Bone remodeling is also fundamental to skeletal restoration in fracture healing (Figure 1F). Although remodeling is often only stated as the final stage of fracture repair, it is required for the transition from the soft to the hard callus,^12^ and subsequent remodeling to a consolidated cortical structure. These sequences of cellular events reflect the same stages of endochondral ossification observed in growth plate and cortical bone development, as the callus is transformed from a cartilaginous to a lamellar bone structure. If the fracture is fixed and there is no cartilage formation, it would only need the sequence where woven bone is remodeled and replaced with lamellar bone. As previously, the mineralized substrate is removed by osteoclasts, followed by osteoblasts appearing and acting on the previously resorbed surface to form a new bone substance, following the same remodeling sequence of activities.^13^

In all situations listed above, bone resorption is followed by bone formation on the same surface. This conserved sequence of activities is bone remodeling. The coupling process, which controls this sequence, is therefore important in many contexts including adult skeletal maintenance, bone growth, cortical and trabecular bone development, and fracture healing.

### Basic concepts: Coupling is unidirectional, asynchronous, local, and sometimes imbalanced

It is important to note that coupling is unidirectional. It describes the communication from the osteoclast to the osteoblast to control this sequence.^5^^,^^6^ As a mnemonic, it can be helpful to note that “coupling” is also used to describe the hooking mechanism by which a locomotive (the osteoclast) is attached to the carriages (the osteoblasts) that follow it. There are, of course, other communication mechanisms in the other direction, from osteoblast lineage cells to osteoclasts. This includes the way osteoblast lineage cells support osteoclast formation through the RANKL/OPG system.^14^^,^^15^ I use the term “coupling” here in its original context to refer to the unidirectional signal from the osteoclast to the bone-forming osteoblasts, thereby controlling the remodeling sequence at a specific location. Perhaps a second term could be found to refer to this second communication mechanism occurring in the opposite direction to avoid further confusion in the literature.

Another critical early theory was that coupling must be controlled locally. This emerged as a way to explain the asynchronous nature by which remodeling occurs throughout the skeleton.^6^ In the same histological section, which represents a single point in time, BMUs were found at different stages of activity. This implied that bone remodeling is not controlled systemically. The authors stated: “*Both resorption and formation processes can be found in all stages of evolution in the same bone at the same time, while being supplied by the same blood.*” ^6^ This would mean the sequence must be primarily controlled at the local cellular level, not by a factor within the circulation. At that time, the field was focused on understanding the skeleton and its control by systemic calcium-regulating hormones, such as PTH and calcitonin (eg, Parfitt^16^). It was a novel concept to suggest local paracrine mechanisms might be important.

Although coupling and remodeling are local events, their impact on the skeleton can be systemic. This is most clearly illustrated in a therapeutic setting. For example, in patients treated with denosumab, osteoclast numbers are reduced.^17^ This means that, throughout the skeleton, which is supplied by the same bloodstream carrying the anti-RANKL therapeutic, fewer osteoclasts initiate resorption and thereby initiate remodeling, resulting in a low bone remodeling rate. Since this occurs throughout the skeleton, it lowers biochemical markers of bone resorption in the circulation.^18^ Since there are fewer remodeling cycles initiated, less coupling signals are produced within the local environment. This means there are fewer bone-forming osteoblasts throughout the skeleton, leading to low levels of serum bone formation markers.^18^

The balance between bone resorption and bone formation within each BMU is an important outcome of the sequence of remodeling, particularly in adult life.^19^ For bone mass to remain the same within a BMU, the amount of bone resorbed by the osteoclasts must be replaced by the subsequent bone-forming activity of osteoblasts. This is not always the case. Although the remodeling sequence occurs locally, it is influenced both by local events at the bone surface and by systemic changes, such as in ageing (including menopause)^20^ or in pathologies like rheumatoid arthritis.^21^ For example, if there is increased bone resorption due to such systemic influences, but osteoblasts do not fully replace the amount of bone removed, the activity is imbalanced. Sometimes this has been referred to as “uncoupling,” but this is not accurate, because the sequence of activities within the BMU is maintained; bone resorption has been followed by bone formation, meaning coupling has occurred. However, resorption and formation are imbalanced; if this occurs in multiple BMUs throughout the skeleton, it leads to bone loss.^20^

Although coupling is a well-established theory, it is not possible to measure coupling itself since it occurs over a passage of time. Coupling activity is inferred based on histomorphometry, which measures an historic record at a single point in time. It can only be accurately inferred based on data from the remodeling surfaces described above, usually limited to cortical cutting cones and trabecular secondary spongiosa. Our current understanding of coupling depends on comparing the proportions of bone surface covered with bone-forming osteoblasts to those with osteoclasts, or those in reversal (ie, with evidence of resorption, but without osteoclasts or bone-forming osteoblasts on the surface). In mouse studies, coupling activities can only be inferred based on osteoblast and osteoclast surfaces, because murine osteoclasts resorb shallow pits; their lack of Howship’s lacunae in normal conditions means that eroded and reversal surfaces cannot be accurately identified. Serum biochemical markers, in preclinical or clinical studies, rarely reflect coupling because they provide a sum of activities throughout the skeleton, including both remodeling and modeling surfaces.

### Mechanisms of coupling I: The reversal phase

Multiple mechanisms have been proposed by which the osteoclast might signal to the bone-forming osteoblast. An important principle is that the communication between these cell types occurs during the intervening period between bone resorption and formation. This is known as the reversal phase, and coupling mechanisms must persist through this period.

When the reversal phase was first described, using a rat model of induced alveolar bone remodeling,^22^ it was reported that there was 3-fold more bone surface in the reversal phase than in the resorption phase. This suggests that the reversal phase duration is longer than the time taken to resorb bone during remodeling. In adult trabecular bone, this was estimated to be approximately 34 d in duration,^23^ though it is likely that this duration varies with different conditions, including age, species, and anatomical location.^24^

The earliest coupling mechanisms proposed were the events of the reversal phase.^25^ Two mechanisms, or their combination, were suggested to be responsible: (1) signals from the cells present during the reversal phase, described at the time as mononuclear phagocytes, or (2) changes to the bone surface during the reversal period (such as deposition of the cement line).

The cement line is a series of modifications made to the bone surface during the reversal phase. It is well known for its function to connect newly formed bone with older bone, and as a mechanism to slow crack propagation in response to strain.^26^ A second proposed function of the cement line is to provide coupling signals. The cement line might use multiple mechanisms to attract osteoblast progenitors. One candidate found at high levels in the cement line is osteopontin,^27^ which is also apparent when the Ploton silver stain is used to mark the lacunocanalicular system.^28^ Osteopontin might act as a chemotactic agent to attract osteoblast lineage cells to the bone surface, thereby providing a localization signal necessary for coupling.^27^ It has also been suggested that demineralized collagen within the cement line may act as a chemoattractant^29^; additional signals deposited in the cement line by osteoclasts or osteoblast lineage cells may remain to be identified.

In the 1990s, in vitro studies suggested that the cement line was not necessary for copuling to occur. Primary calvarial osteoblasts were plated onto dentine slices with a grid of mechanically carved grooves.^30^ Staining with Alizarin red demonstrated that the cultured osteoblasts preferentially formed bone within the grooves. The bone formed within the grooves was more than simply a thin layer of mineral, and by backscattered electron microscopy was confirmed to be 3D bone with osteocyte lacunae. This suggested that osteoclasts, simply by resorbing a pit, can indicate to osteoblasts a location requiring bone formation. This could be the “surface tactic effect,” initially proposed by Frost and colleagues.^6^ This seems to be sufficient to induce localized bone formation by osteoblasts, at least in an experimental system where they are provided in bulk to that location.

It appears that the presence of a resorbed surface is not always sufficient in vivo. In a biopsy study in the early 1980s, it was noted that the extent of reversal phase compared to osteoclast surface was much greater in biopsies from women with postmenopausal osteoporosis.^31^ This led to the proposal that bone formation could not be maintained at levels sufficiently high to match bone resorption in postmenopausal osteoporosis because of an uncoupling of the osteoclast (the locomotive) from the bone-forming osteoblasts (the carriages). Osteoclasts were resorbing bone pits, as indicated by the presence of Howship’s lacunae, but there was a lack of signals for osteoblasts to follow.

Many years later, the identity of the reversal cells was revealed by immunohistochemical studies in human biopsies to include osteoblast lineage cells.^32^ Active reversal surfaces containing osteoblast lineage cells were identified by the presence of CD56 and osterix protein. In contrast, in samples from women with postmenopausal osteoporosis, when elongated uncoupled (or “arrested”) reversal surfaces were observed, there were fewer CD56/osterix-positive cells. This also provided evidence that the pit alone was not sufficient for bone formation to follow resorption in vivo; a further signal, from the reversal cells themselves, was implicated.

**Figure 2:**
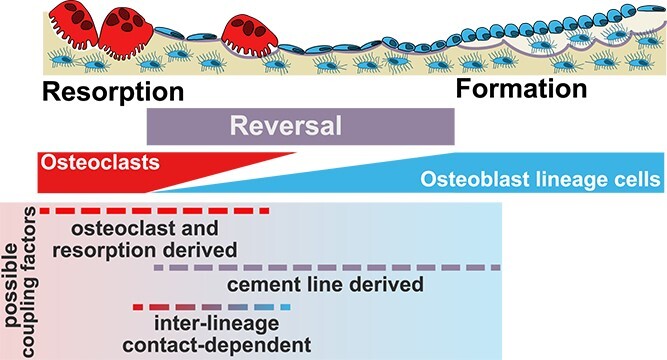
Possible subtypes of coupling signals, which may act during transitions within the reversal phase. During the reversal phase, coupling signals resulting from osteoclast resorptive action or osteoclast secretion, and signals from the cement line are likely to be present at different, but overlapping, periods during the reversal phase. The reversal phase is likely to be a period with multiple phases, including a gradual decline in osteoclast density and a gradual increase in osteoblast lineage cell density, until sufficient cells are present for bone formation to commence on the resorbed surface. This includes a suggested time period when inter-lineage cell-contact-dependent communication between osteoclasts and the osteoblast lineage may occur. For simplicity, only cells on the bone surface are included, although there are likely multiple interactions with other cells in the vicinity of the BMU; see text for further details.

In the same study, tartrate-resistant acid phosphatase-positive cells were also observed within the reversal surface.^32^ This was surprising at the time, because the established model of the remodeling sequence was that osteoclasts, reversal cells, and osteoblasts are present on the bone surface in 3 separate phases (Figure 1A). This observation led the team to suggest progressive stages within the reversal phase,^33^ as follows (Figure 2). At the point of the cutting cone, there is an initial phase of tunneling resorption where osteoclasts are exclusively present. This is followed by a mixed reversal-resorption phase where osteoblast linage cells are intercalated with osteoclasts. Although osteoblast lineage cells are present, bone formation has not yet commenced. During this reversal-resorption phase, there is a gradual accumulation of osteoblast lineage cells until they reach a critical mass with the ability to form bone at the closing end of the cutting cone.^33^ This provides a very different way of thinking about the reversal phase, including multiple opportunities for coupling signals to be transmitted between osteoclasts and osteoblast lineage cells. This would include contact-dependent mechanisms within the reversal-resorption phase and increasing contact between osteoblast lineage cells in the later periods of the reversal phase. The latter could be a signal that leads to matrix production, reflecting early observations that formation of bone-like collagen-containing mineralized nodules in vitro occurs in foci where osteoblasts are most tightly packed.^34^^,^^35^

Another proposed mechanism, active during the reversal phase, which raises many unanswered questions, is that osteocytes might also provide an independent coupling mechanism. This emerged from finite element analysis modeling of increased strain in the areas of bone resorption, both in the cutting cone and in the trabecular BMU.^36^ That analysis suggested that osteocytes might release osteocyte-derived coupling factors in response to the increased strain. This is an area requiring further exploration, but osteocytes produce factors that stimulate bone formation in response to strain, such as STAT3-dependent cytokines.^37^ Their contributions to the sequence of activities during bone remodeling, particularly during the reversal phase, remain to be defined.

### Mechanisms of coupling II: Osteoclast-derived coupling factors

During the reversal phase, there are also signals provided by the osteoclasts themselves that could promote subsequent bone formation at the same location. These signals must persist through the reversal period or operate during the mixed reversal-resorption phase. Osteoclast-derived factors could be released from the matrix by osteoclasts during bone resorption or be produced and expressed by the osteoclasts themselves. These have become known as coupling factors and many mechanisms have been proposed for their activities (recently reviewed in detail, Sims and Martin^38^).

The term “coupling factor” was first proposed in 1981 as a mechanism for how bone resorption and bone formation increased in parallel in organ cultures of embryonic chick tibiae treated with parathyroid hormone (PTH).^39^ This theoretical model for how osteoclasts might communicate with bone-forming osteoblasts has become useful even though those cultures did not contain bone; an embryonic chick tibia is still cartilaginous.^40^ The team suggested that coupling factors could be produced by the osteoclast, or could be released by bone resorption,^41^ and would then act on osteoblast progenitors or osteoblasts themselves to stimulate bone formation at the same location as the prior resorption.

This raised many questions, most importantly, the identity of that factor or factors, and led to an expansion of work using in vitro systems to begin identifying candidates. The first genre of candidate molecules were those discovered by purifying non-collagenous proteins from the bone matrix, such as bone morphogenetic protein (BMP),^42^ or from media in which bones were cultured, such as the transforming growth factors (TGFs).^43^ This led to the suggestion that osteoclasts, while resorbing bone, would release these factors from the matrix into the local environment. When activated, they would recruit osteoblasts or their progenitors toward the resorbed bone surface to enable bone formation at that location. There remain many questions about how this might work: how are levels controlled, given the variable content of each of these factors in different regions of bone? What concentrations would be released? Would these factors remain locally for long enough for the osteoblast precursors to find them, migrate to the surface, and form bone? At which points in the reversal phase would they act? Are they relevant to physiology or pathological conditions?

Shortly after this, further in vitro studies indicated that osteoclasts could also synthesize factors capable of stimulating osteoblast differentiation. Some early examples were TGFβ^44^ and hepatocyte growth factor.^45^ In separate experiments, these proteins induced bone formation in organ culture^46^ or stimulated proliferation when applied to cultured osteoblast-like cells.^45^ The same questions were raised about how these factors, like those released by osteoclast activity, could work within the BMU.

Another type of coupling factor identified was one that would promote chemotaxis of osteoblast progenitors. These too were identified through in vitro studies, with sphingosine-1-phosphate being one of the first. Stimulation of bone marrow macrophages with RANKL (receptor activator of NF-kappa B ligand) to form osteoclasts induced sphingosine-1-phosphate production. When sphingosine-1-phosphate was then applied to osteoblast-like cells, their chemotaxis was stimulated, and this was blocked with a specific inhibitor.^47^

The idea arose that coupling factors would need to act in multiple ways on osteoblast progenitors.^48^ Osteoclasts would release multiple factors either from the bone matrix, or by synthesizing them. The factors would then act, but not on mature bone-forming osteoblasts, because they would not yet be present in the BMU. Instead, they would act on skeletal stem cells to recruit them, to induce their migration, or to stimulate their commitment to become osteoblasts. It was their migration to the specific location that was most important to provide those cues as to where bone formation should happen. This would require coordination of both location-dependent and location-independent mechanisms. For example, the osteoclasts might release factors that attract the pluripotent precursors, with additional signals, perhaps provided by reversal cells, the cement line, or osteocytes. These latter signals, all located within the resorption pit, could provide the location signals for osteoblast lineage cells to migrate appropriately and form bone where needed.

### Evidence for osteoclast-derived coupling factors in vivo

With the advent of genetically altered mice, in vivo studies began to shed light on how coupling factors might act. In early work from our laboratory, we studied mice with very low trabecular bone volume due to a presumably coupled elevation in both osteoclast and osteoblast numbers.^49^ This model had elevated signaling of one pathway downstream of glycoprotein 130 (gp130), the common receptor subunit used by the interleukin 6 (IL-6) family of cytokines. When primary calvarial osteoblasts from these mice were cultured, there was no increase in their differentiation. This suggested that the increase in bone formation observed in vivo was secondary to coupling signals from the osteoclasts*.* When these mice were crossed with mice lacking IL-6 to test its involvement in the process, their low bone mass phenotype was worsened, because osteoclast numbers remained higher than controls, but there was no corresponding increase in osteoblasts. We suggested that this might reflect an uncoupling of resorption and formation. This suggested that the coupling factor being produced in the gp130 mutant mice was IL-6 dependent, though we believed it was unlikely to be IL-6 itself.^50^

At around the same time, it was proposed that osteopetrotic mice and humans (with high bone mass due to impaired bone resorption) might provide a useful insight into coupling factor mechanisms.^50-52^ This arose from the recognition that there are 2 forms of osteopetrosis. In “osteoclast-poor” osteopetrosis (for example, where there is RANKL or c-fos deficiency), osteoclasts are absent because they cannot be generated. In this form of osteopetrosis, bone formation is low due to the lack of initiation of remodeling, as described above in the context of denosumab therapy. In contrast, in “osteoclast-rich” osteopetrosis, osteoclast function is defective, but osteoclast numbers are maintained, or sometimes increased. Although bone resorption was impaired in “osteoclast-rich” osteopetrosis, bone formation, and therefore the ability of osteoblasts to follow osteoclasts, was maintained. This was exciting because it coincided with the development of Cathepsin K inhibitors as potential anti-resorptive therapies for osteoporosis. When Cathepsin K inhibitors were used in rabbit^53^ and monkey studies,^54^ there was less osteoclast resorptive activity, but since osteoclasts remained present, bone-forming activity of osteoblasts was maintained and in some instances increased. This suggested that the ability of osteoclasts to signal to osteoblasts to form bone on the resorbed surface did not depend on osteoclast’s resorptive activity; osteoclasts could produce coupling factors independent of resorption.

### Identifying coupling factors—the burden of proof

In the years following this, many coupling factors have been proposed (see an extensive table in^38^). Mechanisms have been proposed where coupling factors could act early (during the initiation of the resorption phase) during the resorptive phase where they’re released from the resorbed matrix, synthesized by the osteoclast, or perhaps released as micro-vesicles, all of which act on osteoblast progenitors.

The evidence that these are truly coupling factors is patchy and these studies all have limitations, and the activity of these candidates within the BMU is not limited to their proposed role in coupling. It is possible to test some of these ideas in in vitro systems, but it needs to be acknowledged that these provide an idealized situation. For example, a recombinant protein can be added to cultured osteoblast-like cells until the concentration is reached where markers of osteoblast differentiation are changed in the desired way, but whether this represents the concentrations osteoblast progenitors are exposed to in vivo is not known.

Although many factors have been proposed to be candidate coupling factors, it is important to define what evidence is sufficient to support this idea. I suggest there are 3 important milestones. First, it is necessary to show that the factor is produced or released by the osteoclast in vivo*,* preferably in multiple species. This requires localization information. Next, functional data must show that these factors can stimulate bone formation in vivo, not just stimulate osteoblast-like qualities in cell culture. Ultimately, in vivo evidence of a connection between the osteoclast and the bone-forming activity of the osteoblast is needed. An example of this might be induced cell-specific genetic deficiency of the candidate in osteoclasts, leading to a phenotype of resorption that is not followed by bone formation. This is a challenge for the field because no coupling factor proposed to date has achieved this level of evidence.

### Cardiotrophin 1: An osteoclast-derived coupling factor with multiple actions within the osteoblast lineage

To explore some limitations of studies designed to identify coupling factors, I give an example from our own work, a cytokine called cardiotrophin 1 (CT-1). We found by immunohistochemistry that CT-1 was localized in murine osteoclasts actively resorbing bone.^55^ Through in vitro studies of a stromal cell line, we found that recombinant CT-1 could dose-dependently stimulate markers of osteoblast differentiation at the expense of adipogenesis.^55^ When mice were injected with recombinant CT-1 locally, we observed an increase in bone formation,^55^ although it must be recognized that this could have been an idealized dose, greater than what the osteoblasts are usually exposed to.

When we explored mechanisms by which CT-1 might promote bone formation, we found 2 potential pathways. Recombinant CT-1 acted on progenitors to stimulate transcription factors related to early osteoblast commitment (C/EBPδ and Runx2),^55^ and inhibited sclerostin expression in cultured osteocyte-like cells.^56^ These qualities are shared with other cytokines of the IL-6 family also proposed as coupling factors, such as Oncostatin M and Leukemia inhibitory factor.^56^^,^^57^ This suggested that the osteoclast-derived CT-1 might signal not only to osteoblast progenitors, but might also act on osteocytes to release a brake on bone formation (sclerostin), which could then promote bone formation in a location-specific manner. In the context of coupling, we know no mechanism by which CT-1’s action on osteocytes could be delayed sufficiently after the osteoclasts had completed their resorptive function, nor which of these mechanisms might be at play within a BMU.

When CT-1 knockout mice were studied, it was possible to infer a reduction in coupling activity, because there was a greater osteoclast surface than controls, but no corresponding elevation in osteoblast surface^55^: bone resorption was separated from bone formation. Although we could not measure eroded or reversal surfaces, because they cannot be observed in mice, we could detect impaired resorption by the presence of cartilage remnants within the trabecular bone. This meant that the CT-1 deficient osteoclasts had a functional defect in their resorptive capacity, and the knockout mice were an example of “osteoclast-rich” osteopetrosis. Whether the inability of osteoblasts to keep up with osteoclasts was due to the loss of CT-1 secretion from the osteoclasts or due to reduced osteoclast activity and subsequent loss of matrix-derived coupling factors cannot be resolved.

### EphrinB2: An example of a candidate coupling factor with multiple actions in the BMU

Another early coupling factor proposed was EphrinB2, and this was particularly intriguing because it was proposed to act through a contact-dependent mechanism.^58^ EphrinB2 is a membrane-bound protein, and has been localized by immunohistochemistry in murine and rat osteoclasts in tissue sections.^58^^,^^59^ When its receptor (EphB4) was overexpressed in osteoblasts in vivo, using the *Col1a1* promoter, there was a high bone mass phenotype and a high level of bone formation.^58^ This suggested that EphrinB2 from the osteoclast could act on osteoblasts to promote bone formation. However, EphrinB2 from osteoclasts was not essential, because when EphrinB2 was knocked out in the myeloid lineage there was no phenotype detected: no change in bone mass or in osteoblast numbers.^58^ If this were an important coupling factor, this would have been expected.

What was perplexing at the time was that the coupling mechanism proposed was contact-dependent. This was suggested before the mixed reversal-resorption stage had been observed. It was difficult to imagine that there would be direct contact of osteoclasts with osteoblasts, or even with osteoblast progenitors.

The role of EphrinB2 as a coupling factor was also complicated by the presence of EphrinB2 in other cells within the BMU. EphrinB2 is also expressed in the osteoblast lineage,^59^ which require cell–cell contact to produce bone matrix. When the interaction of EphrinB2 with its receptor was blocked pharmacologically, osteoblast differentiation was impaired, both in vitro^59^ and in vivo*.*^60^ Later work showed that this defect in osteoblast differentiation might be explained by an anti-apoptotic function of EphrinB2 / EphB4 signaling, which we confirmed in *Osterix-Cre-*driven EphrinB2-deficient mice.^61^ This broadened the role of EphrinB2 in bone to include effects within the osteoblast lineage independent of osteoclasts. When EphrinB2 deletion was targeted to late osteoblasts and osteocytes using *Dmp1Cre*, the resulting mice displayed normal bone structure, but reduced bone strength and greater brittleness due to an increase in bone matrix mineralization.^62^

This provides an example of how a single putative coupling factor may have multiple effects within the same BMU. In the case of EphrinB2, when osteoclasts resorb bone at the start of the remodeling sequence, there is no contact between the osteoclast and osteoblast lineage cells; since osteoclasts do not express EphB4, and EphrinB2 has not been observed in the cellular canopy overlying the BMU, there is no role for EphrinB2/EphB4 signaling during this part of the remodeling process. It is possible that EphrinB2/EphB4 osteoclast-mediated coupling might occur within the early reversal-resorption phase, where there are opportunities for direct contact between osteoclasts and osteoblast progenitors. Later in the reversal phase, as osteoblast progenitors build up on the bone surface and make extensive cell–cell contact during bone formation, the interaction of EphrinB2 and EphB4 within the osteoblast lineage becomes important to prevent apoptosis and thereby promotes bone formation. Finally, at the very end of the remodeling sequence, when osteoid has been deposited and mineralization has commenced, EphrinB2/EphB4 signaling within the connected osteoblasts and the osteocyte network inhibits mineralization and maintains the flexibility of bone.

### Integration of signals, redundancy, and potential situational specificity

The above example of EphrinB2 is but one of many proposed coupling factors within the BMU that are expressed in multiple cell types and have multiple actions during the remodeling sequence; these have been tabulated in an earlier review.^38^ This raises another question to be resolved: how do these factors interact with the multiple cell types present at different stages in the BMU? There are more than just osteoclasts and osteoblast lineage cells in the BMU microenvironment; cells such as endothelial cells, macrophages, and T cells, which express many of the proposed osteoclast-derived coupling factors, are present and would also contribute to osteoblast differentiation. This has been discussed at length elsewhere.^63^

Why would there be so many different factors and mechanisms that control this sequence of events? It could be that some of them have been mis-identified. They may not be coupling factors at all but have other roles in promoting bone formation, perhaps in pathologies where there are more osteoclasts bringing osteoblasts or their progenitors to the bone surface. Given the importance of the coupling process, redundancy may be required to maintain bone mass in the absence of a single factor. This redundancy may also explain why a sustained anabolic response to treatments for osteoporosis is so difficult to achieve.

It is also probable that the coupling process, like the step-by-step processes controlling osteoclast differentiation, involves multiple levels of control at multiple stages of osteoblast differentiation: some signals to promote precursor migration, some working at stages during the differentiation process, and others that provide information about the location where bone is to be formed. This would include early interactions between the osteoclast and the canopy of osteoblast lineage cells that sit between the resorption pit and the marrow cavity,^64^ explored in detail previously.^38^ Later signals, after coupling, would provide information about how much bone is required; osteocytes are ideally placed for this, since they could sense when the level of strain caused by the earlier resorption has been relieved by adequate bone formation.

There may be environment-dependent, substrate-specific, and species-specific coupling factors. As discussed above, remodeling in the adult skeleton, in endochondral ossification, and in fracture healing may each involve coupling factors and mechanisms specific for these processes. For example, an in situ hybridization study of human adolescent bone detected neither CT-1 nor EphrinB2 in osteoclasts,^65^ in contrast with earlier immunohistochemical studies in mouse and rat bone.^55^^,^^58^^,^^59^ This may reflect species, age, or anatomical differences in these candidate coupling factors. An example of substrate-dependent factors could be that, in endochondral ossification, resorption of cartilage at the hypertrophic zone would be expected to release different matrix-derived products, the cement line on a cartilaginous surface would likely have a different composition, and osteoblast precursors would exist in a different microenvironment to that of trabecular bone. This warrants further exploration.

## Summary, conclusions, and future directions

In summary, coupling is a local mechanism, which controls a sequence of activities at a specific location. Coupling includes multiple mechanisms produced by multiple cell types, and these mechanisms must bridge the period between bone resorption and subsequent bone formation, including the reversal phase. There are many candidate coupling factors proposed, but none have been conclusively shown to play a role.

There are many questions remaining, not limited to the identity and specificity of coupling factors. These include: how is reversal phase duration controlled and could shortening the reversal phase provide an approach to anabolic therapy? What signals from reversal cells and the cement line contribute to coupling? Do osteocytes produce specific factors in response to the microscopic strains produced by bone resorption during remodeling? Is it possible, using new imaging techniques, to visualize and record the sequence of coupling? Do substrate-, region-, or age-specific coupling factors exist? Are there factors specific to pathological processes, or at specific stages of reproduction? Could we provide a coupling factor to stimulate bone formation without stimulating resorption for an anabolic effect or to augment or replace current anabolic therapies? Clearly, there is much more work to be done to understand this process fully.
